# Variability in PET image quality and quantification measured with a permanently filled ^68^Ge-phantom: a multi-center study

**DOI:** 10.1186/s40658-023-00551-w

**Published:** 2023-06-16

**Authors:** O. Sipilä, J. Liukkonen, H.-L. Halme, T. Tolvanen, A. Sohlberg, M. Hakulinen, A.-L. Manninen, K. Tahvanainen, V. Tunninen, T. Ollikainen, T. Kangasmaa, A. Kangasmäki, J. Vuorela

**Affiliations:** 1 HUS Diagnostic Center, Clinical Physiology and Nuclear Medicine, Helsinki University Hospital and University of Helsinki, P. O. Box 442, 00029 Helsinki, Finland; 2 Radiation and Nuclear Safety Authority, Vantaa, Finland; 3 Turku PET Centre, Turku University Hospital, Turku, Finland; 4 Department of Nuclear Medicine, Päijät-Häme Central Hospital, Lahti, Finland; 5 Department of Clinical Physiology and Nuclear Medicine, Diagnostic Imaging Center, Kuopio University Hospital, Kuopio, Finland; 6 Department of Applied Physics, University of Eastern Finland, Kuopio, Finland; 7 OYS Department of Nuclear Medicine and Radiology, Oulu University Hospital, Oulu, Finland; 8 Medical Research Center Oulu, Oulu University Hospital and University of Oulu, Oulu, Finland; 9 Department of Clinical Physiology and Nuclear Medicine, Satakunta Central Hospital, Pori, Finland; 10 Clinical Physiology and Neurophysiology, North Karelia Central Hospital, Joensuu, Finland; 11 Department of Clinical Physiology and Nuclear Medicine, Vaasa Central Hospital, Wellbeing Services County of Ostrobothnia, Vaasa, Finland; 12 Department of Imaging and Radiotherapy, Docrates Cancer Center, Helsinki, Finland; 13 Clinical Physiology and Nuclear Medicine, Central Finland Health Care District, Jyväskylä, Finland

**Keywords:** PET-CT, Recovery coefficient, Image quality, ^68^Ge NEMA/IEC phantom

## Abstract

**Background:**

This study evaluated, as a snapshot, the variability in quantification and image quality (IQ) of the clinically utilized PET [^18^F]FDG whole-body protocols in Finland using a NEMA/IEC IQ phantom permanently filled with ^68^Ge.

**Methods:**

The phantom was imaged on 14 PET-CT scanners, including a variety of models from two major vendors. The variability of the recovery coefficients (RC_max_, RC_mean_ and RC_peak_) of the hot spheres as well as percent background variability (PBV), coefficient of variation of the background (COV_BG_) and accuracy of corrections (AOC) were studied using images from clinical and standardized protocols with 20 repeated measurements. The ranges of the RCs were also compared to the limits of the EARL ^18^F standards 2 accreditation (EARL2). The impact of image noise on these parameters was studied using averaged images (AVIs).

**Results:**

The largest variability in RC values of the routine protocols was found for the RC_max_ with a range of 68% and with 10% intra-scanner variability, decreasing to 36% when excluding protocols with suspected cross-calibration failure or without point-spread-function (PSF) correction. The RC ranges of individual hot spheres in routine or standardized protocols or AVIs fulfilled the EARL2 ranges with two minor exceptions, but fulfilling the exact EARL2 limits for all hot spheres was variable. RC_peak_ was less dependent on averaging and reconstruction parameters than RC_max_ and RC_mean_. The PBV, COV_BG_ and AOC varied between 2.3–11.8%, 9.6–17.8% and 4.8–32.0%, respectively, for the routine protocols. The RC ranges, PBV and COV_BG_ were decreased when using AVIs. With AOC, when excluding routine protocols without PSF correction, the maximum value dropped to 15.5%.

**Conclusion:**

The maximum variability of the RC values for the [^18^F]FDG whole-body protocols was about 60%. The RC ranges of properly cross-calibrated scanners with PSF correction fitted to the EARL2 RC ranges for individual sphere sizes, but fulfilling the exact RC limits would have needed further optimization. RC_peak_ was the most robust RC measure. Besides COV_BG_, also RCs and PVB were sensitive to image noise.

## Background

Positron emission tomography (PET) measures quantitative information on the distribution of a radioactive tracer in a patient, presented as activity concentration (Bq/ml) in the images. These measurements are used to compute standard uptake values (SUVs) by normalizing them with patient weight (or lean body mass) and injected activity [1]. The SUVs are commonly utilized for classifying abnormal tracer uptake as benign or malignant, as well as for follow-up of disease progress [2–5]. Thus, besides visual image quality, measured activity concentration should not significantly vary between PET scanners in which the patient might be imaged. Moreover, reference SUV values for disease stages should be reliably utilizable in all PET scanners. In practice, the choice of technical settings including imaging, image reconstruction and post-processing parameters could account up to 55% variability in the measured activity concentration [6]. In addition, variations in practical implementation including patient preparation may impact the result of the imaging study.

To test the performance characteristics of a PET scanner, the metrics in National Electrical Manufacturers Association (NEMA) NU 2 standards have been widely adopted, e. g. [7–10]. The newest version of this standard was published in 2018 [11]. To facilitate multicenter quantitative imaging studies, several programs and software tools for harmonizing recovery coefficients (RCs) of activity concentration in small hot objects have been implemented [12–16]. The RC is defined as the ratio of the activity concentration measured from the PET image to the known activity concentration of the object. Most commonly, the hot spheres in the NEMA/International Electrotechnical Commission (IEC) NU2 image quality (IQ) phantom [11] have been utilized for the RC measurements. Several definitions for RCs exist including maximum, mean and peak RC values [17]. Calibration of the activity meter (dose calibrator) utilized for cross-calibration of the PET scanner (e. g. [18]) as well as PET image noise and resolution may have strong influence on the RC values [19].

Besides hot object contrast, image noise and cold object contrast have a major effect on visual image quality and thus on valid interpretation of the image. Widely utilized PET IQ parameters include coefficient of variation of the background voxel values (COV_BG_) [12, 18, 20], percent background variability (PBV) [11] and accuracy of corrections (AOC) [11]. Noise equivalent count rate (NECR) has been studied for noise level optimization of patient images, e. g. [21, 22], although with the modern iterative reconstruction methods the results have been quite variable. Moreover, radiomics models, as e. g. in [23], might be utilized for estimating IQ features directly from the clinical images.

When utilizing relatively short-lived PET isotopes, often ^18^F with a half-life of 1.8 h, separate filling of a phantom is usually required for every measurement session, and the measurement time is limited due to the decay of the activity. Thus, the variance in the measurement results may be influenced by the differences in phantom filling processes and in activity measurements. To avoid these limitations, NEMA IQ phantoms permanently filled with a relatively long half-life isotope of ^68^Ge (271 d) have been utilized to study e. g. repeatability and reproducibility of serial PET measurements [24], noise and signal properties of reconstructions including point spread function (PSF) correction [25] as well as feasibility of using them in IQ assessment in multicenter clinical trials [26, 27].

In our work, a NEMA IQ phantom permanently filled with ^68^Ge was imaged in Finnish PET centers to study differences in quantification and in image quality. The 14 PET scanners included in the study varied from older models without PSF correction or time of flight (TOF) available to digital systems from two major vendors. All scanners had integrated computed tomography (CT). Variations in RCs as well as in IQ parameters measurable with the NEMA IQ phantom, including COV_BG_, PBV and AOC, were evaluated from the routinely used whole-body imaging protocols of each PET center and from standardized protocols. The ranges of RC values, proportional to the ranges of SUV values among Finnish PET centers, were evaluated, as well as the comparability of these ranges to the limits of EARL ^18^F standards 2 accreditation [28], which are referred to as EARL2 limits in the rest of the article. In addition, the impact of image noise on the RC and IQ results was studied.

## Material and methods

### Phantom imaging

A NEMA 2018 IQ phantom with ^68^Ge was imaged in 11 Finnish PET centers during June 2019–January 2020. The total activity of the phantom varied from 30.6 to 17.5 MBq. The measurements were performed using 14 PET-CT scanners, including analog and digital systems from two major vendors (Tables 1, 2). The phantom included six hot spheres with diameters of 10, 13, 17, 22, 28 and 37 mm. The activity concentration ratio of the spheres to the background was 4:1. In addition, a cold lung insert was included.

**Table 1:** Scanners and imaging parameters for routine protocols 1r–14r

Protocol number	Scanner			Routine protocol								
	Scanner	Software version	Axial detector length (cm)	Slice thickness (mm)	Pixel size (mm)	Matrix	Reconstruction	Iterations/subsets	Filter or β-factor	Phantom imaging time (s)	Phantom activity concentration (MBq/kg)	Background bias correction factor*
1r	Siemens Biograph mCT Flow	VG60A	21.6	5	2.04	400	OSEM, TOF + PSF	2/21	XYZ Gauss5,00	103–168	2.8–1.8	0.96
2r	Siemens Biograph mCT Flow	VG60A	21.6	5	3.18	256	OSEM, TOF + PSF	2/21	XYZ Gauss4,00	136	2.9	0.94
3r	Siemens Biograph Horizon Flow	VJ20B	22.1	5	4.11	180	OSEM, TOF + PSF	5/10	XYZ Gauss4,00	136	2.9	0.94
4r	Siemens Biograph mCT Flow	VG60A	21.6	3	3.18	256	OSEM, TOF + PSF	2/21	XYZ Gauss3,00	152	2.9	0.92
5r	Siemens Biograph Vision 600 Flow	VG75B	26.3	3	1.65	440	OSEM, TOF + PSF	4/5	All-pass	152	2.9	0.92
6r	Siemens Biograph mCT	VG60A	16.2	3	4.07	200	OSEM, TOF + PSF	2/21	XYZ Gauss2,00	123	2.9	1.11
7r	Siemens Biograph TruePoint	PET/CT 2009A	16.4	5	4.07	168	OSEM	4/8	XYZ Gauss5,00	204	2.6	0.96
8r	GE Discovery 690	52.00	15.7	3.27	3.65	192	OSEM, TOF + PSF	2/24	Cutoff 6.4 mm, *z*-axis standard	126	3.0	0.94
9r	GE Discovery 690	52.00	15.7	3.27	3.65	192	OSEM, TOF + PSF	2/24	Cutoff 6.4 mm, *z*-axis standard	112	3.2	0.95
10r	GE Discovery MI	pet_col_bb.31	20	2.79	2.73	256	Q.Clear	–	150	112	3.2	0.93
11r	GE Discovery MI	pet_col_bb.31	20	2.79	2.73	256	Q.Clear	–	150	126	3.0	0.98
12r	GE Discovery MI	pet_col_bb.31	25	2.8	2.73	256	Q.Clear	–	150	122	3.0	0.98
13r	GE Discovery MI	pet_col_bb.31	20	2.79	2.73	256	OSEM, TOF + PSF	3/16	Cutoff 5.0, *z*-axis standard	100–115	2.8–2.4	0.94
14r	GE Discovery STE (mobile installation)	41.04	15.7	3.27	5.47	128	3D IR	2/20	Cutoff 5.0, *z*-axis standard	253	2.1	0.91

^*^Mean from 20 images with SDs of 0.002–0.008

**Table 2:** Standard protocols 1s–13s

Protocol number	Standard protocol			
	Slice thickness (mm)	Pixel size (mm)	Iterations/subsets	Background bias correction factor*
1s	3	2.74	2/21	0.95
2s	3	2.74	2/21	0.92
3s	3	2.49	4/10	0.93
4s	2.03	2.74	2/21	0.91
5s	3	2.36	8/5	0.91
6s	3	2.74	2/21	1.10
8s	3.27	2.73	3/16	0.94
9s	3.27	2.73	3/16	0.95
10s	2.79	2.73	3/16	0.91
11s	2.79	2.73	3/16	0.97
12s	2.8	2.73	3/16	0.96
13s	2.79	2.73	3/16	0.93

The numbering refers to the same scanners as in Table 1. Standard protocol was not available for scanners 7 and 14. All routine protocols used OSEM reconstruction, TOF and PSF correction. The matrix size was 256 × 256

^*^Mean from 20 images with std of 0.000–0.006

#### Routine protocols

In every PET-CT scanner, the local clinical imaging protocol for the whole body [^18^F]FDG studies was used. CT was used for attenuation correction. The main parameters of the 14 protocols are listed in Table 1. The routine protocols were numbered as 1r–14r. PET imaging was repeated 20 times during the same imaging session, except for protocol 14r there was only 10 repetitions due to technical reasons. In addition, imaging session for protocol 1r was repeated five times and protocol 13r three times during a period of 5.5 months for estimating the impact of intra-scanner variations on the measurements.

**Fig. 1:**
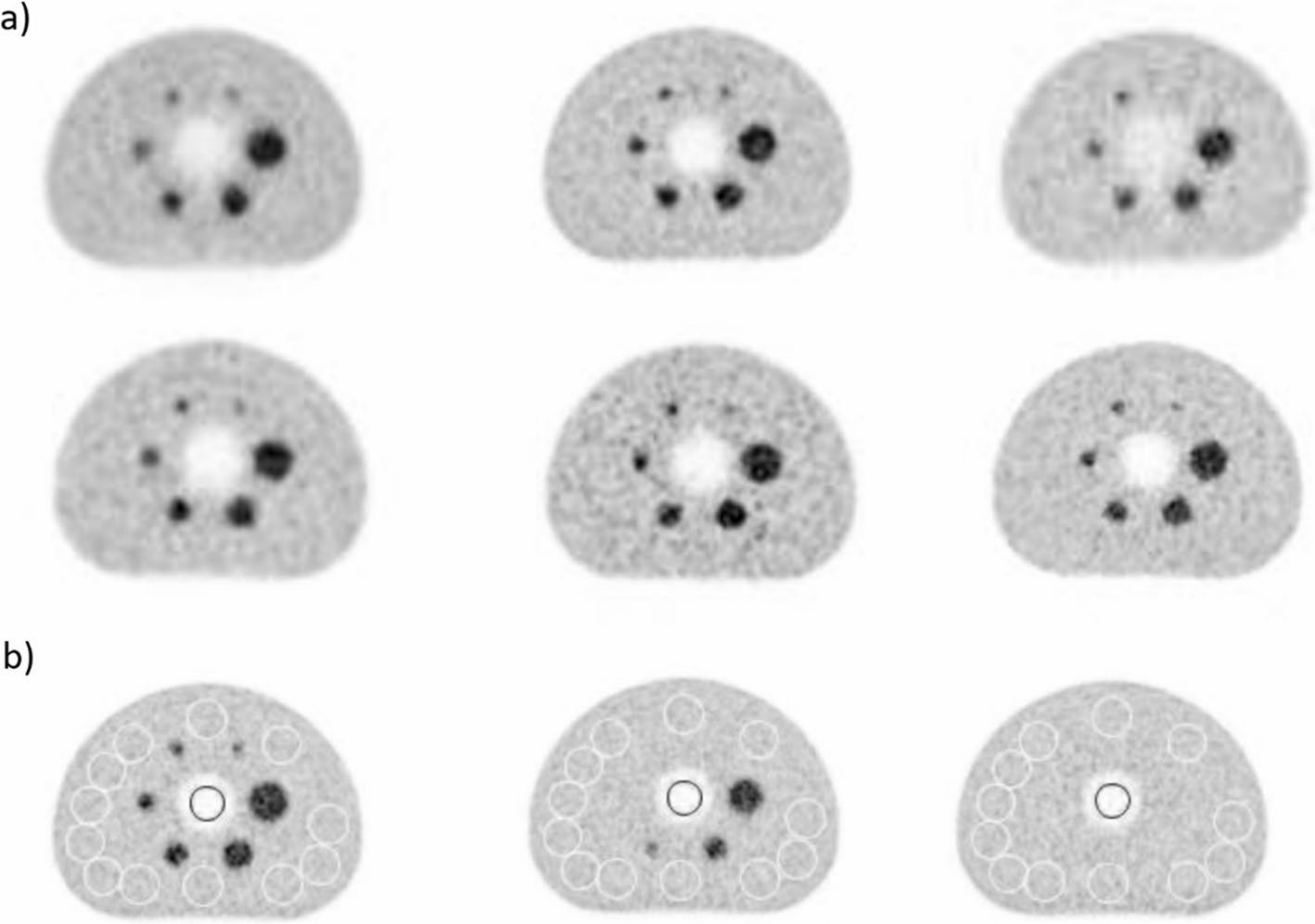
Examples of phantom images and ROIs. **a** Axial plane from the middle of the six hot spheres in the NEMA IQ phantom filled with ^68^Ge as imaged with six different scanners using the local routine protocols. The signal void in the middle of the slice is from the cylindrical lung insert utilized in the AOC test. **b** Axial plane from the middle of the six hot spheres (left image) as well as one centimeter (middle image) and two centimeters (right image) from it. The locations of the NEMA specified background ROIs with a diameter of 37 mm are shown with the white circles. Besides the 36 out of 60 background ROIs shown here, the rest of the ROIs were situated in the planes of one and two centimeters before the middle plane. The background ROIs with smaller diameters (10, 13, 17, 22 and 28 mm) were centered inside the 37 mm ROIs. The ROI with a 30 mm diameter inside the lung insert is shown in black in these planes

The imaging time for each session was adjusted according to the average activity concentration of the phantom in the imaging day, varying from 3.2 to 1.8 MBq/kg (Table 1). In addition, the imaging time of the phantom was linearly scaled according to the clinically used patient activity concentration (MBq/kg) of the whole body [^18^F]FDG studies in the particular PET center. The scaling also included the effect of slightly variable patient resting times utilized in different centers. The goal was to preserve the differences in the relative count rates of routine imaging protocols between the centers with different optimization strategies and scanners available. The scaled imaging times are also listed in Table 1. The time-activity-product (TAP) of the routine protocols varied between 4.6 and 9.0 min*MBq/kg. In scanners with stationary bed positions, two positions were imaged with the overlapping region placed in the middle of the hot spheres of the phantom. In Fig. 1a, axial slices in the middle plane of the hot spheres from six different routine protocols are shown.

#### Standard protocols

In addition, with scanners enabling PSF correction, the phantom was imaged 20 times with standardized imaging parameters. The standard protocols were numbered as 1s–6s and 8s–13s. The number is referring to the same scanner as in the routine protocols. Two of the scanners (7 and 14) did not enable PSF correction. In the standard protocols, only one bed position with the middle plane in the middle of the hot spheres was imaged. The imaging time was 5 min for the one bed position, or the corresponding bed speed was utilized in scanners with continuous bed motion. The images were reconstructed using ordered-subsets expectation–maximization (OSEM), TOF, PSF correction, matrix size of 256 × 256 and no filtering. Scatter, random and dead time corrections were enabled. The (number of iterations) * (number of subsets) as well as the slice thicknesses and pixel sizes were standardized as accurately as possible. The slight variations in these parameters can be found in Table 2.

### Data processing

Image analysis was conducted using in-house developed automated MATLAB scripts (R2019b; The MathWorks, Inc., Natick, Massachusetts, USA).

#### Data sets

As every PET imaging protocol was repeated 20 (or 10) times during an imaging session, the analyses were performed 20 (or 10) times and the final result was reported as the mean value of these 20 (or 10) repetitions. In addition, an average image (AVI) of the 20 (or 10) repeated PET images was computed to simulate a very low noise image to be utilized in some of the analyses.

#### Coefficient of variation (COV)

Coefficient of variation was used to compare the results from different data sets. It was computed as the standard deviation of the results divided by the mean of them and multiplied by 100%.

#### Background bias correction

To exclude the impact of the cross-calibration of the scanner and/or calibration of the activity meter utilized in an individual PET center, images were corrected for the calibration biases before further analysis, if not stated otherwise. For the correction, a background bias correction factor (BBCF) was computed as the mean phantom background value from the PET image divided by the time corrected true activity concentration in the phantom background as stated in the calibration certificate of the phantom. The mean phantom background value was computed as the mean voxel value of the 60 background regions of interests (ROIs) with a 37 mm diameter (C_B, 37 mm_) utilized also in the NEMA IQ analysis [11] (Fig. 1b).

#### Recovery coefficients (RCs)

To compute an RC, the maximum, mean or peak activity concentration [17] of a hot sphere was measured from a PET image and divided by the known activity concentration. The maximum activity concentration was computed as the maximum voxel value in the volume of interest (VOI) including all voxels inside a hot sphere. The mean activity concentration was computed as the mean voxel value in the VOI including voxels with values ≥ 50% of the maximum voxel value inside a hot sphere. The peak activity concentration was computed as the highest mean value in a spherical VOI with a diameter of 12 mm and the center voxel inside a hot sphere.

For all sphere sizes, the computed RCs from the 20 (or 10) repeated PET series were averaged to obtain the final RC_max_, RC_mean_ and RC_peak_ values. The corresponding RC values were also computed from the AVIs.

#### Comparison of the RCs in the routine protocols

To estimate the range in SUV values between scanners and protocols routinely used in Finnish PET centers, the maximum range of RC_max_, RC_mean_ and RC_peak_ values from the routine protocols were computed without background bias correction.

Intra-scanner variability vs. inter-scanner variability for the RC_max_, RC_mean_ and RC_peak_ was studied by comparing the mean COV of the corresponding RCs from the five scanning sessions of protocol 1r and the three scanning session of protocol 13r to the mean COV from all 14 different protocols (1r – 14r). The mean COVs were computed as the mean of the COVs for the six different sized hot spheres.

#### Comparison of the routine and standard protocols to EARL2 limits

Routine and standard protocols with PSF correction and BBCF with variation of < 10% from the nominal value of 1 were included in the comparison to EARL 2 accreditation limits for the RC_max_, RC_mean_ and RC_peak_ [28], which can be found in Table 3. The ranges of the maximum and minimum limits for the RC_max_, RC_mean_ and RC_peak_ are also tabulated. The number of the routine and standard protocols as well as the corresponding AVIs fulfilling the EARL2 limits was reported. Moreover, the number of RC results fulfilling the EARL limits for an individual sphere size was counted. It was also checked for the individual spheres, whether the range of the RCs fitted to the range of the corresponding EARL2 limits (but not necessarily the exact upper and lower limits of EARL2).

**Table 3:** Upper and lower limits of RCs and their ranges in EARL2 [26]

	Diameter of the hot sphere (mm)					
	10	13	17	22	28	37
RC_max_						
Upper	0.88	1.22	1.38	1.32	1.26	1.29
Lower	0.52	0.85	1.00	1.01	1.01	1.05
Range (upper–lower)	0.36	0.37	0.38	0.31	0.25	0.24
RC_mean_						
Upper	0.61	0.86	0.97	0.99	0.97	1.00
Lower	0.39	0.63	0.76	0.80	0.82	0.85
Range (upper–lower)	0.22	0.23	0.21	0.19	0.15	0.15
RC_peak_						
Upper	0.41	0.70	0.99	1.10	1.10	1.10
Lower	0.27	0.45	0.75	0.90	0.90	0.90
Range (upper–lower)	0.14	0.25	0.24	0.20	0.20	0.20

Besides direct comparison of the RCs to the EARL2 limits and ranges, the COVs of the RC_max_, RC_mean_ and RC_peak_ from the included protocols were computed for each six differently sized hot sphere. The mean value of the COVs for all six spheres (meanCOV_max_, meanCOV_mean_, meanCOV_peak_) was reported as a measure of the similarity of the RC values of the included protocols. In addition, the mean values of the RC_max_, RC_mean_ and RC_peak_ for all sphere sizes for a single protocol (MCR, as in Ref. [29]) was computed, and the COVs of these MCRs (COVMCR_max_, COVMCR_mean_, COVMCR_peak_) were reported as a measure of the similarity of the shape of the RC curves.

#### PBV and COVBG

For the routine and standard protocols and AVIs, the PBVs for each sphere diameter *j* were computed as the *N*_*j*_s in the corresponding NEMA 2018 test [11]

1 $${N}_{j}=\frac{{\mathrm{SD}}_{j}}{{C}_{B,j}}*100\%,$$

where *C*_*B,j*_ is the average value of the voxel values in the *K* (= 60) circular background ROIs with diameter *j* (10, 13, 17, 22, 27 and 37 mm) and SD_*j*_ the standard deviation of the average values of the *K* individual background ROIs with diameter *j*

2 $${\mathrm{SD}}_{j}=\sqrt{\sum_{k=1}^{K}{\left({C}_{B,j,k}-{C}_{B,j}\right)}^{2}/(K-1)}$$

To estimate the effect of averaging on *N*_*j*_, the results from the single images were divided by the results from the AVIs for each diameter *j*. In addition, for each protocol the effect of the diameter was reported as the ratio of the maximum and minimum *N*_*j*_.

The results from the routine protocols were also compared to the 10% limit required by the Finnish Radiation and Nuclear Safety Authority [30].

COV_BG_ was computed using voxel values from 60 circular background ROIs with the diameter of 37 mm specified in the NEMA IQ test [11] for the routine protocols and AVIs. The results were compared to the 15% limit used as a criterium for sufficient clinical image quality in Refs. [31, 32].

#### Accuracy of corrections (AOC)

As in the NEMA Accuracy of Corrections test, Δ*C*_lung,*i*_ was first computed for every slice *i* in the axial range of the lung insert of the phantom and excluding those slices nearer than 30 mm from the axial edges of the insert [11]:

3 $$\Delta {C}_{\mathrm{lung}, i}=\frac{{C}_{\mathrm{lung}, i}}{{C}_{B, 37\mathrm{ mm}}}*100\%$$

*C*_lung,*i*_ was the mean voxel value of a circular ROI with a diameter of 30 mm inside the lung insert in slice *i* and *C*_*B*_, 37 mm the mean voxel value of the 60 background ROIs with a diameter of 37 mm (Fig. 1b). The final accuracy of corrections (AOC) was computed as a mean of Δ*C*_lung,*I*_ from all slices i.

## Results

### Background bias correction

The BBCFs varied between 0.91 and 0.98, except for protocols 6r and 6s the factors were 1.11 and 1.10, respectively (Tables 1 and 2).

### Comparison of the RCs in the routine protocols

**Fig. 2:**
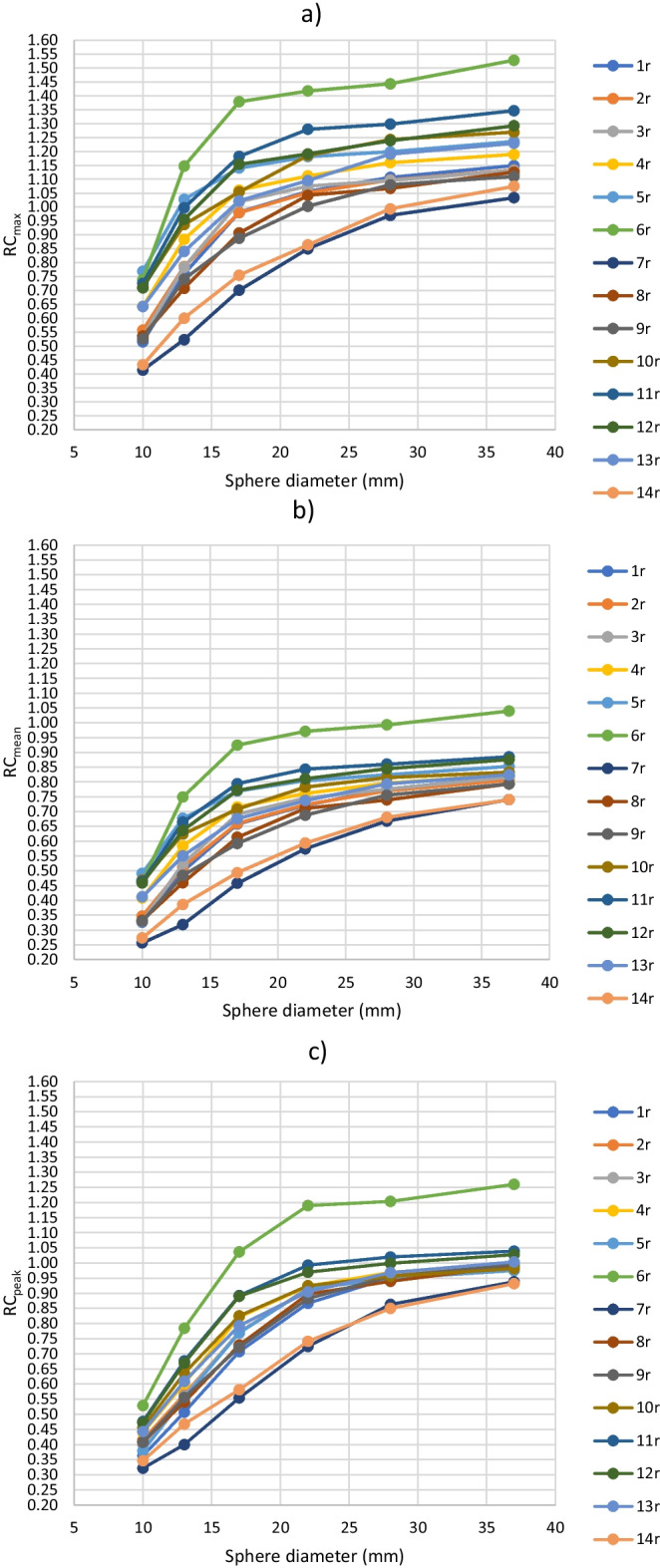
**a** RC_max_, **b** RC_mean_ and **c** RC_peak_ computed from the hot spheres with the diameter of 10–37 mm using routine protocols 1r–14r without background bias correction

The RC_max_, RC_mean_ and RC_peak_ values for routine protocols 1r–14r without background bias correction are presented in Fig. 2. The maximum ranges of the RC_max_ values were 0.36, 0.62, 0.68, 0.57, 0.47 and 0.49 for the sphere sizes of 10 mm, 13 mm, 17 mm, 22 mm, 28 mm and 37 mm, respectively. The corresponding ranges for the RC_mean_ were 0.23, 0.43, 0.47, 0.40, 0.32 and 0.30, and for the RC_peak_ 0.21, 0.38, 0.48, 0.47, 0.35 and 0.33. When computing the RC_max_, RC_mean_ and RC_peak_ values, the biggest SD from averaging the 20 (10) individual results for each protocol and sphere size was 0.15.

The inter-scanner mean COV of the RC_max_ values from the routine protocols 1r–14r was 15.3%. The corresponding intra-scanner mean COVs were 1.8% and 1.6% for protocols 1r and 13r, respectively. For the RC_mean_, the inter-scanner mean COV was 15.3%, and the intra-scanner mean COV was 1.4% for both protocols 1r and 13r. For the RC_peak_, the corresponding inter-scanner value was 12.3% and intra-scanner values were 0.7% and 1.0%. Thus, the intra-scanner mean COVs were about 10% of the corresponding inter-scanner mean COVs and about 10% the inter-scanner variabilities could be accounted to repeatability issues of different measurement sessions.

### Comparison of the routine and standard protocols to EARL2 limits

**Fig. 3:**
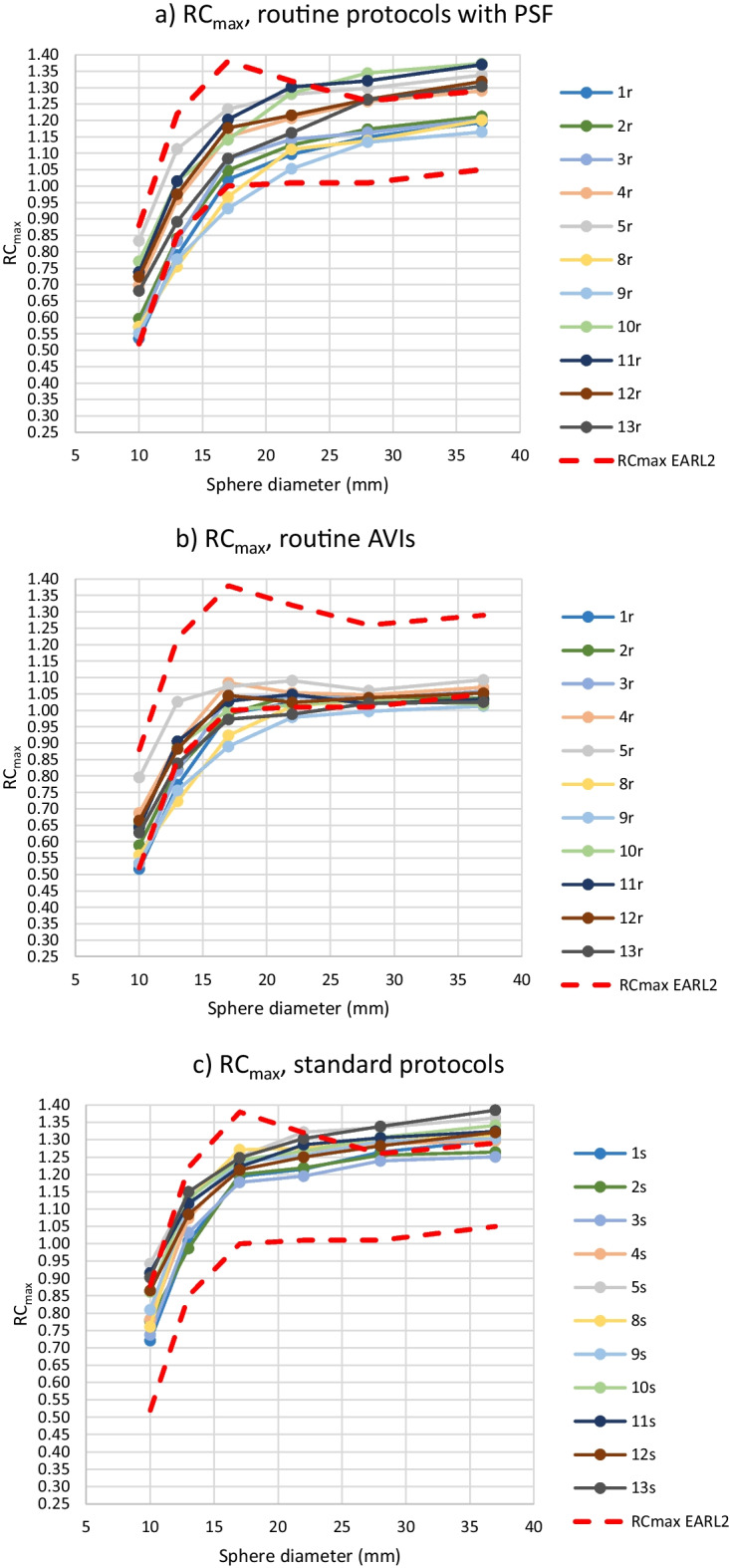

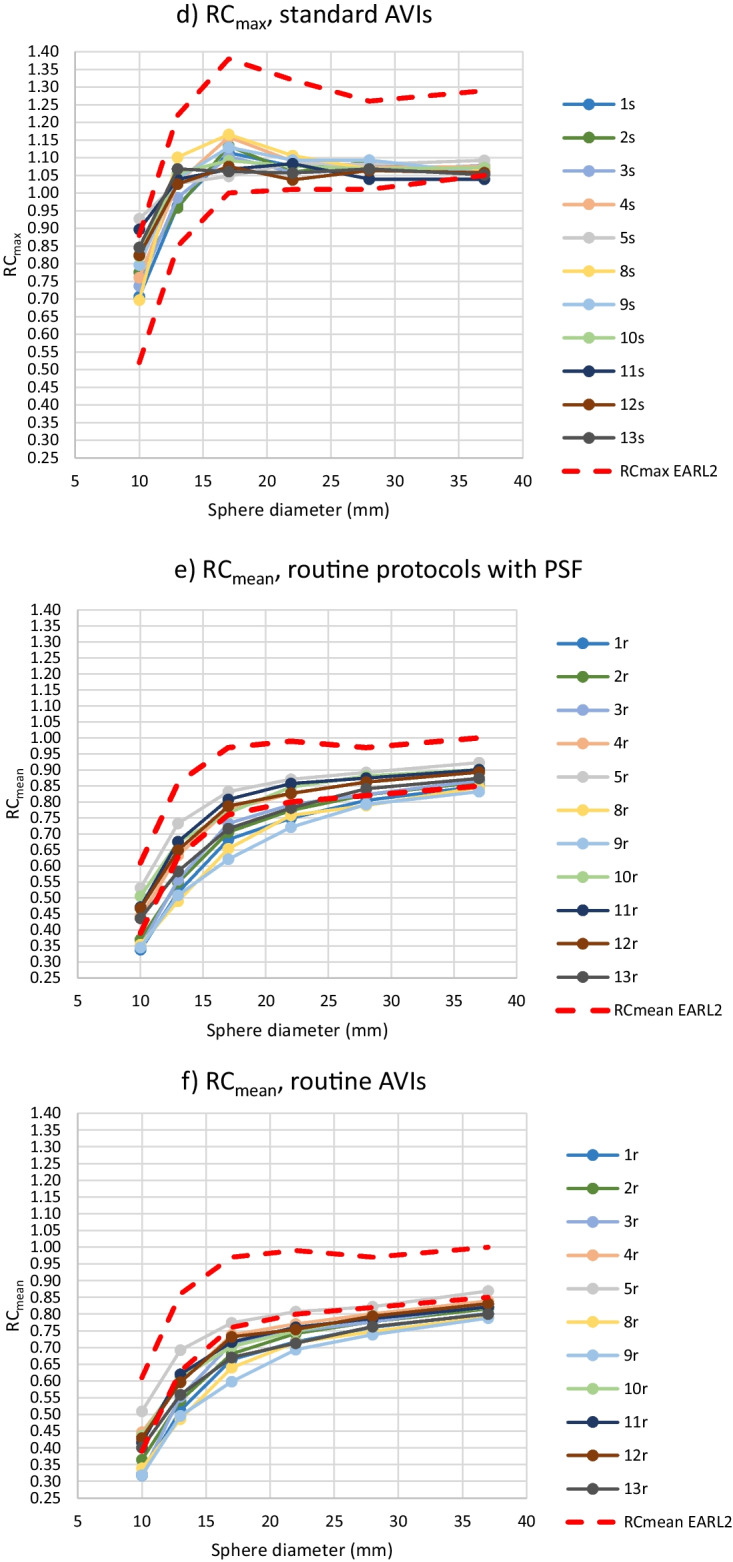

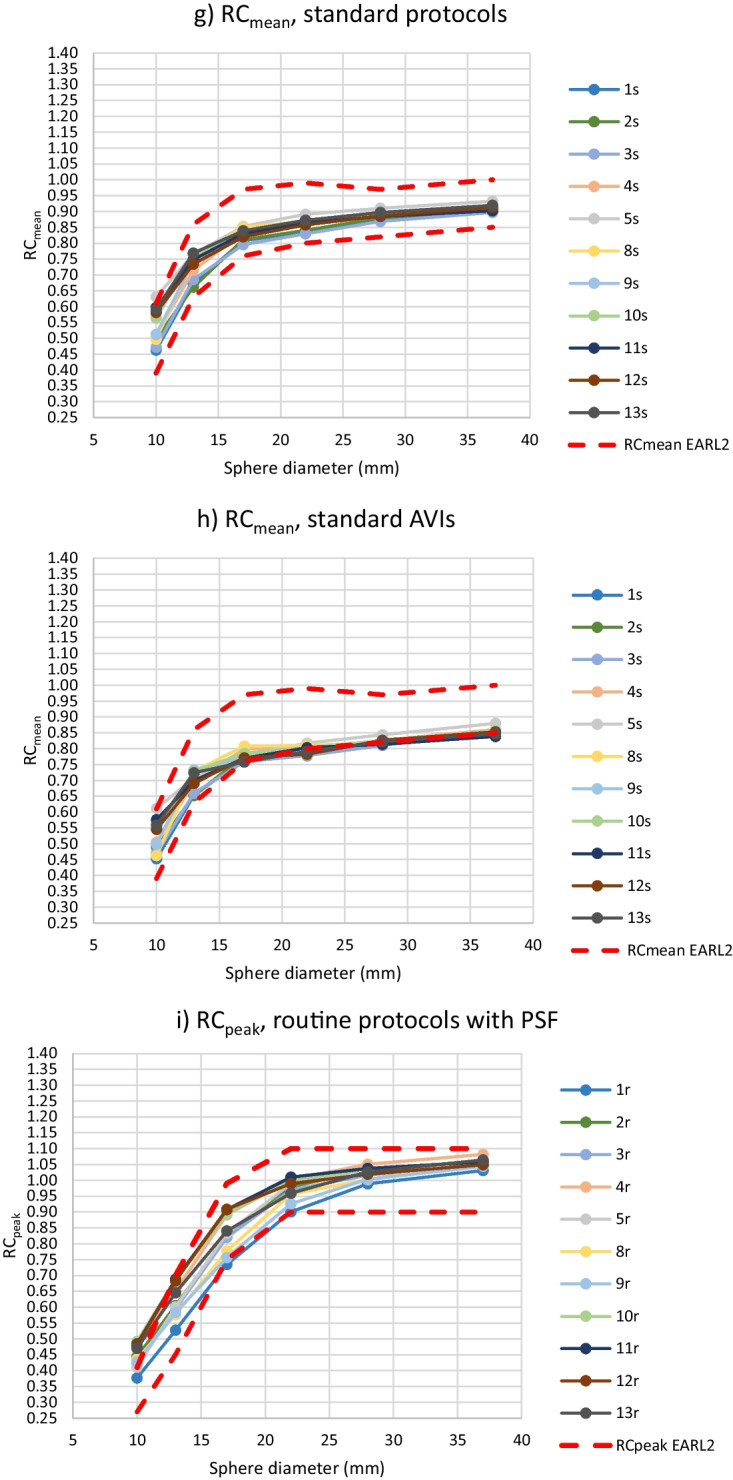

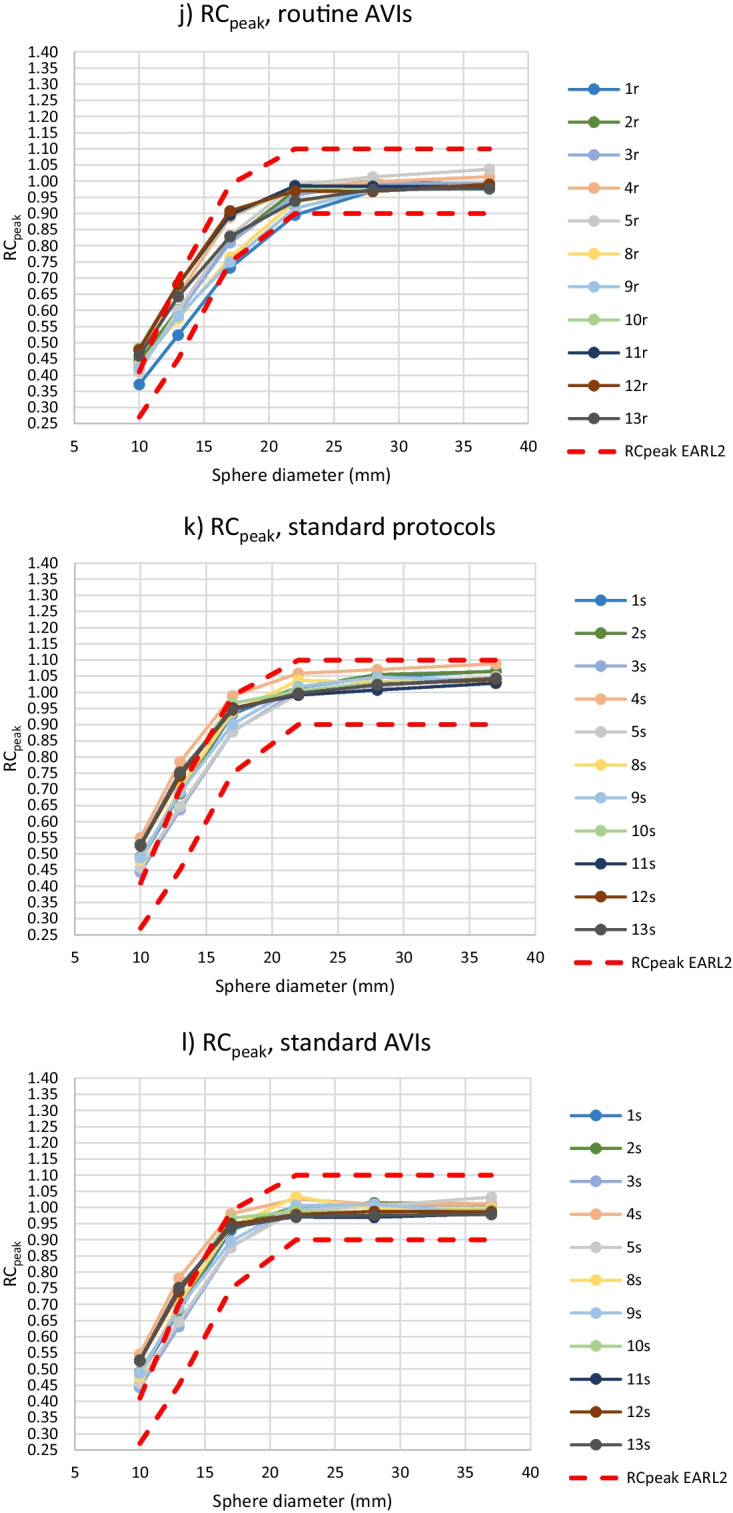
RC_max_ (**a**–**d**), RC_mean_ (**e**–**h**) and RC_peak_ (**i**–**l**) computed from the hot spheres with the diameter of 10–37 mm using routine protocols with PSF correction (**a**, **e**, **i**) and the corresponding AVIs (**b**, **f**, **j**) and standard protocols (**c**, **g**, **k**) and the AVIs (**d**, **h**, **l**). Protocols 6r and 6s were excluded. The upper and lower limits of EARL2 are also shown in the images

From further analysis of the RC values, routine protocols 6r, 7r and 14r and standard protocol 6s were excluded, because the BBCFs of protocols 6r and 6s were more than 10% over the nominal value and protocols 7r and 14r did not include PSF correction. For the rest of the 11 routine and standard protocols and corresponding AVIs, RC_max_, RC_mean_ and RC_peak_ values are presented in Fig. 3. For the 11 routine protocols, the maximum ranges of the RC_max_ values were 0.30, 0.36, 0.30, 0.25, 0.21 and 0.21 for the sphere sizes of 10 mm, 13 mm, 17 mm, 22 mm, 28 mm, and 37 mm, respectively. The corresponding ranges for the RC_mean_ were 0.19, 0.24, 0.21, 0.15, 0.10 and 0.09, and for the RC_peak_ 0.12, 0.16, 0.17, 0.11, 0.06 and 0.05. The percentages of the protocols fulfilling the EARL limits for all six sphere sizes as well as separately for the individual spheres are presented in Table 4.

**Table 4:** The percentages of the routine and standard protocols and AVIs fulfilling the limits of the EARL2 of RC_max_, RC_mean_ and RC_peak_ for all the six sphere sizes as well as separately for individual sphere sizes. In addition,  the meanCOVs and COVMCRs of the different protocols are listed

	Percentage of protocols fulfilling EARL2 limits for all six spheres (%)	Percentage of individual spheres fulfilling EARL2 limits (%)	meanCOV (%)	COVMRC (%)
RC_max_				
Routine protocols	0	73	9.4	8.5
Routine AVIs	27	67	6.1	4.9
Standard protocols	18	67	4.2	3.5
Standard AVIs	82	96	3.6	1.3
RC_mean_				
Routine protocols	45	56	8.8	7.6
Routine AVIs	9	17	7.4	6.1
Standard protocols	91	98	3.7	2.9
Standard AVIs	18	73	3.4	2.1
RC_peak_				
Routine protocols	0	83	5.1	4.1
Routine AVIs	9	82	5.1	3.7
Standard protocols	0	74	3.7	2.6
Standard AVIs	0	74	3.7	2.0

The range of the RC results for individual sphere diameters from all 11 routine or standard protocols or AVIs fitted into the range of the EARL 2 limits in almost 100% of the cases. The only exceptions were the ranges of the RC_mean_ values from spheres of the sizes of 13 and 17 mm in the routine protocols exceeding the corresponding EARL ranges by 5.4 and 0.7%, respectively.

The results for the meanCOVs and COVMCRs can be found in Table 4.

### PBV and COV_BG_

**Fig. 4:**
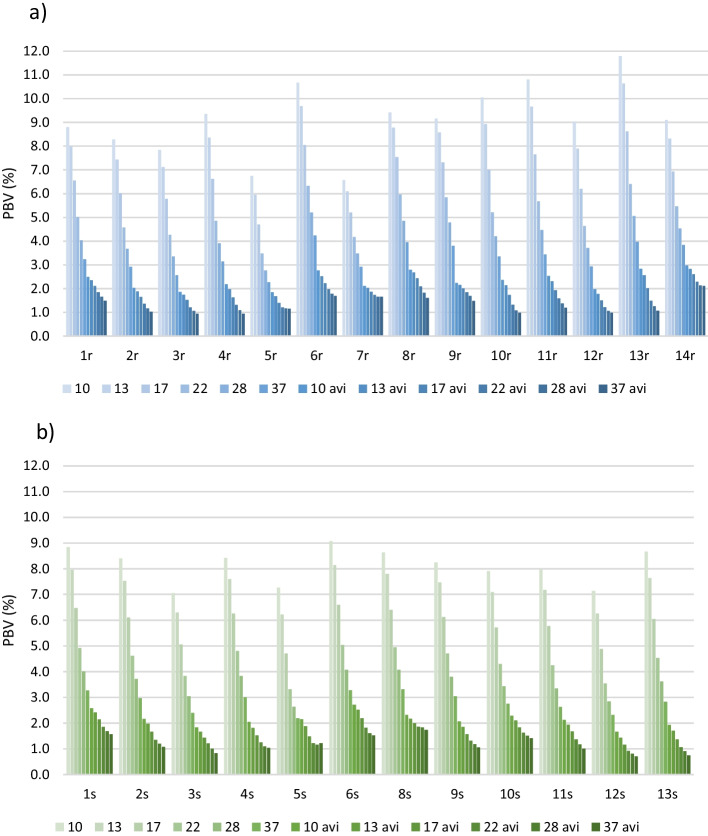
PBV of background ROIs with diameter of 10–37 mm for **a** the routine protocols and AVIs and **b** the standard protocols and AVIs

The PBV varied between 0.9 and 11.8% for the routine images and AVIs (Fig. 4a) and 0.7–9.1% for the standard images and AVIs (Fig. 4b) depending on the ROI size and averaging. The variability for the same sized ROIs was 1.8–4.5 and 2.0–4.6 times smaller in routine and standard AVIs than in the corresponding results computed from single images (routine and standard protocols), respectively, with bigger changes for smaller ROI sizes.

When computing the ratio of the maximum and minimum *N*_*j*_ for each protocol, the maximum value was always found for the diameter of 10 mm and the minimum for the diameter of 37 mm, as can also be observed from Fig. 4. For all the protocols including AVIs, the ratio of the maximum and the minimum values varied between 1.3 and 3.3.

Every routine protocol had *N*_*j*_ less than 10% for ROIs with diameter 17 mm or more. With diameter of 13 mm, one protocol exceeded slightly the 10% limit (10.6%). With the smallest diameter of 10 mm, four routine protocols exceeded the 10% limit (10.1–11.8%).

**Fig. 5:**
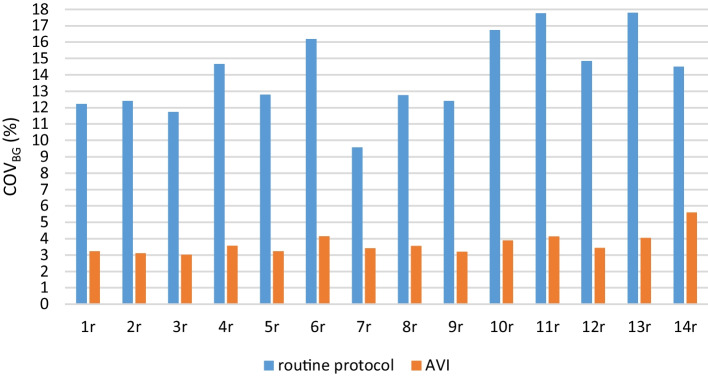
COV_BG_ for all routine protocols (1r–14r) and corresponding AVIs

The COV_BG_ values for the routine protocols and AVIs varied between 9.6–17.8% and 3.0–5.6%, respectively (Fig. 5). In four routine protocols, the COV_BG_ exceeded 15%. If the AVI of protocol 14r with only 10 averaged images was excluded, the maximum COV_BG_ for the AVIs was 4.1%.

### Accuracy of corrections (AOC)

**Fig. 6:**
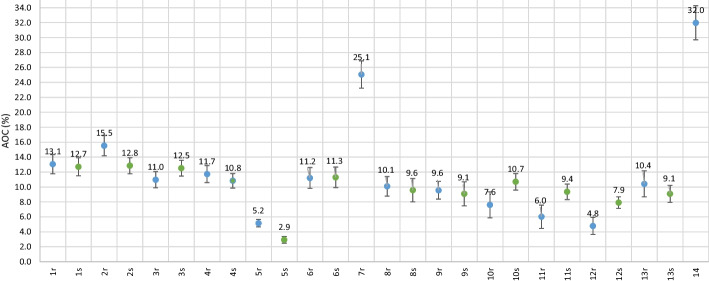
AOC for all routine (blue circles) and standard (green circles) protocols. The mean and standard deviation of Δ*C*_lung,I_ (Eq. 3) from all slices *i* in the NEMA specified spatial range are shown

The AOCs are presented in Fig. 6 for both the routine and standard protocols. For all routine protocols, the AOC varied between 4.8–32.0% with SDs of 0.5–2.4%. When excluding routine protocols without PSF correction, the maximum AOC dropped to 15.5%. For the standard images, the AOCs ranged between 2.9–12.8% with SDs of 0.5–1.7%.

## Discussion

In this study, a NEMA 2018 IQ phantom permanently filled with ^68^Ge was imaged in almost all Finnish PET centers, including a variety of scanner models from two major vendors. After decay correction, the phantom had the same activity concentrations in every measurement, thus excluding the uncertainty of filling the phantom separately for every measurement session. In addition, long measurement sessions with several repetitions were possible. The phantom was imaged with the routine whole-body [^18^F]FDG imaging protocol of each PET center, as well as with a standardized protocol if the scanner enabled PSF correction. The variability in the results of the activity concentration measurements of the small hot spheres as well as image quality parameters measurable with the NEMA IQ phantom were studied.

When using the routine protocol of each PET center, the greatest RC difference without background bias correction was 0.68 for the RC_max_ of the 17 mm sphere, the range being 0.70–1.38. Thus, if taking into account the intra-scanner variability of about 10%, SUV_max_ for a similar-sized small object could range about 60% for the routine whole-body protocols used in the Finnish PET centers due to the variability in the imaging protocols and scanner properties. As can be noticed from Fig. 2, the RC results of protocol 6r, which had a divergent BBCF from the other routine protocols, and the RC results of protocols 7r and 14r without PSF correction expectedly deviated from the rest. When excluding protocols 6s, 6r, 7r and 14r, the RC_peak_ values had smaller ranges than the RC_max_ and RC_mean_ in every sphere size. Similar more robust behaviour of RC_peak_ has also been noticed e. g. in Ref. [33].

When excluding protocols 6r, 6 s, 7r and 14r, the rest of the protocols fitted into the RC ranges of EARL2 in every sphere size, except for two minor exceptions. Majority of the spheres in different protocols also fulfilled the EARL2 upper and lower limits for an individual sphere size, but fulfilling the EARL limits for all sphere sizes of a protocol was scarcer. Thus, it seemed that the ranges of the EARL2 upper and lower RC limits for a sphere size were wide enough to include the results from properly calibrated scanners with PSF correction in the imaging protocol, without any further optimization of the imaging parameters. However, the shape of the RC curves did not necessarily match that of the EARL2 requirements, depending at least on overall averaging (imaging time), the cut-off frequency in filtering and possibly on dissimilarity of other parameters (Fig. 3). It could also be observed that the RC_peak_ curves were less dependent on these factors, especially on the overall averaging, than the RC_max_ and RC_mean_ curves. On the other hand, the shape of the RC_max_ curves was the most dependent on the overall averaging. In this study, the overshoot of RC_max_ values for sphere sizes 13 mm and 17 mm was not so emphasized as in the EARL2 limits.

As can be observed from Table 4, the similarity of RCs (meanCOV_max_, meanCOV_mean_, meanCOV_peak_) as well as the shape of the RC curves (COVMCR_max_, COVMCR_mean_, COVMCR_peak_) were improved by the standardization of the imaging parameters as well as lowering the overall noise level. Still, these changes did not necessarily improve the fulfillment of the exact RC limits defined by the EARL organization. A practical approach for reaching the required shapes of the RC curves would probably be changing the cut-off frequency in filtering during reconstruction as necessary, instead or in addition to standardization and lowering overall noise level. This approach has been suggested e. g. in Refs. [13, 14], with adjusting the cut-off frequency for SUVs on the fly without changing the visual image quality. With the meanCOV and COVMRC results, RC_peak_ seemed again to be the most robust measure among the RCs.

In the PBV test, most of the variability N_j_ seemed to be due to image noise, as the variability dropped with increasing the ROI size and was minimized in the AVIs (Fig. 4). It should be noticed, that the AVI for protocol 14r had only 10 averaged images while the others had 20, probably affecting the result. Besides image noise, the PBV may have reflected spatial variation of the noise, which can be due to the iterative reconstruction methods and corrections utilized [34]. The routine and corresponding standard protocols could not be directly compared, because the imaging time in the standard protocols was longer.

Annual measurement of PBV is also required by STUK with an acceptance level of 10% [30], although it is not specified whether the requirement concerns all sizes (j) of the background ROIs. Using low noise images (AVIs), the 10% limit could be achieved for every ROI size in every protocol used in this study.

As expected, the COV_BG_ values of routine protocols depended mostly on the noise, with 3–4 times smaller values when using AVIs. Part of the COV_BG_ values was probably due to the background variability. The parameters of routine protocols as well as the generations of scanners were quite diverse. Besides imaging time and sensitivity of the scanner, the voxel sizes (8.2–97.8 mm^3^), reconstruction methods and parameters and filtering had distinctive differences, which were reflected in the image noise and thus in the COV_BG_ results.

The COV_BG_ results from the routine and standard protocols could not be compared because of the different imaging times. In addition, the imaging time was the same (5 min) for every standard protocol regardless of the activity of the phantom.

EARL considers COV_BG_ of 15% or smaller to be an acceptable noise level for clinical image interpretation [31]. Some of the routine protocols in our study produced COV_BG_ values exceeding the 15% threshold, which might suggest increasing slightly imaging time in these protocols. As all exceeding results were from scanners with fixed bed positions, the overlapping region of the bed positions with smaller sensitivity may have contributed to the local noise level, as found in Refs. [17, 35].

The AOC results seemed to depend mostly on the generation of the scanner, with better results for protocols with PSF correction available, which has also been observed in other studies, e. g. in Ref. [8]. As can be seen in Fig. 6, the best results were obtained for the newest digital scanners.

There were some non-optimal protocol or phantom related issues in our study that should be noticed when reviewing the results. Although in the routine protocols the imaging and reconstruction parameters as well as the imaging time were chosen to mimic the whole body [^18^F]FDG protocols and timing practicalities clinically used in each individual PET center, the results of the phantom experiments cannot be directly applied to patient studies. The equivalency between count rates in patient and phantom studies cannot be claimed due to different photon flux environments [36, 38]. Moreover, data processing and corrections by a PET system may not have been fully comparable due to different isotopes (^18^F vs. ^68^Ge, which decays through ^68^ Ga) [39]. On the other hand, only small differences in RCs and IQ parameters were found using ^18^F and ^68^ Ga in Ref. [37], and the use of ^68^Ge-filled NEMA IQ phantoms for IQ assessment in multicenter clinical trials has been successfully demonstrated in Refs. [26, 27].

Due to the materials used in the phantom, exact homogeneity of the known activity concentrations in every part of the phantom could not be guaranteed. Especially possible inhomogeneities in the background activity concentration may have had an impact on the PBV and COV_BG_. Instead, the possible structures and relative activity concentrations were the same in every imaging session. The results from the PBV and AOC tests could not be directly compared to results from the corresponding NEMA NU2 2018 tests, since the scatter phantom required to be placed next to the IQ phantom in the NEMA setup was not available in our measurements and the imaging time was not defined according to the NEMA standard [11].

Relating to the computation of the peak activity concentration, the volume used for averaging was bigger than the smallest hot sphere in the phantom. Thus, better PET scanner capabilities, e. g. resolution, might not have been reflected as more truthful RC_peak_ value of the smallest hot sphere.

In this study, no long-term information was assessed. A snapshot of the variations in RCs and IQ accumulated from different sources was obtained, and thus factors affecting stability of the results, such as drifting of an activity meter or calibration of a PET scanner [38, 39], have not been considered.

In conclusion, the largest ranges of the RC (and thus SUV) values of small hot objects due to differences in PET scanners, imaging protocols and parameters was found to be 68%, of which about 10% can be accounted to intra-scanner variability between imaging sessions. The largest ranges were found in the RC_max_ values. The RC ranges from properly calibrated scanners with PSF correction fitted to the EARL2 RC ranges for individual sphere sizes. However, fulfilling the exact upper and lower RC limits and especially the shape of the RC curves would have needed further optimization of the imaging parameters, e. g. cut-off frequency in filtering, in most of the image sets included in this study. RC_peak_ was found to be less dependent on the noise level in the image as well as on other variations in the imaging parameters than RC_max_ and RC_mean_. Most of the RC and IQ results in this study were sensitive to image noise. Thus, if the purpose of the phantom tests were to estimate the performance of PET in clinical use, the image noise level of the clinical protocol should be preserved when choosing the imaging parameters, e. g. imaging time.

## Data Availability

The data analyzed during the current study are available from the corresponding author on reasonable request.

## Abbreviations

AOC: Accuracy of corrections
AVI: Averaged image
BBFC: Background bias correction factor
COV: Coefficient of variation
COV_BG_: Coefficient of variation of the background voxel values
CT: Computed tomography
EARL2: EARL ^18^F standards 2 accreditation
IEC: International Electrotechnical Commission
IQ: Image quality
NECR: Noise equivalent count rates
NEMA: National Electrical Manufacturers Association
OSEM: Ordered-subsets expectation–maximization
PBV: Percent background variability
PET: Positron emission tomography
PSF: Point spread function
RC: Recovery coefficient
ROI: Region of interest
SUV: Standard uptake value
TAP: Time-activity-product
TOF: Time of flight
VOI: Volume of interest
